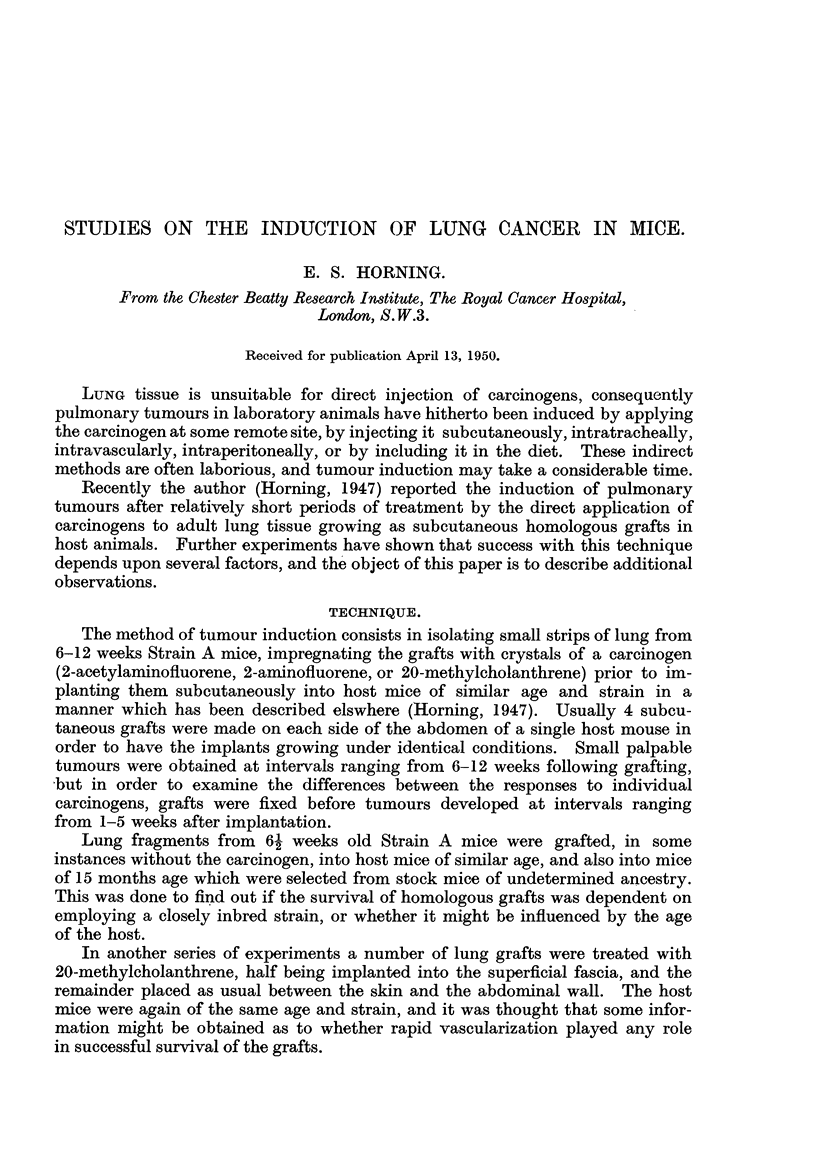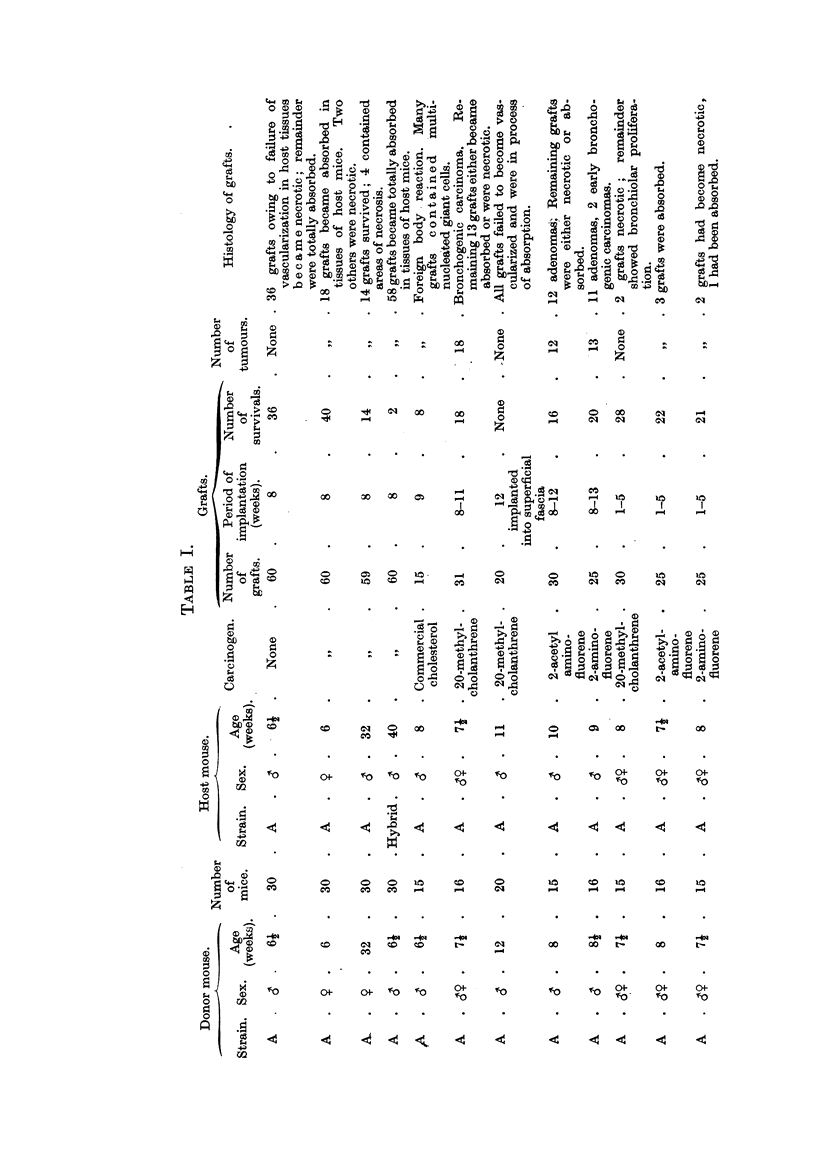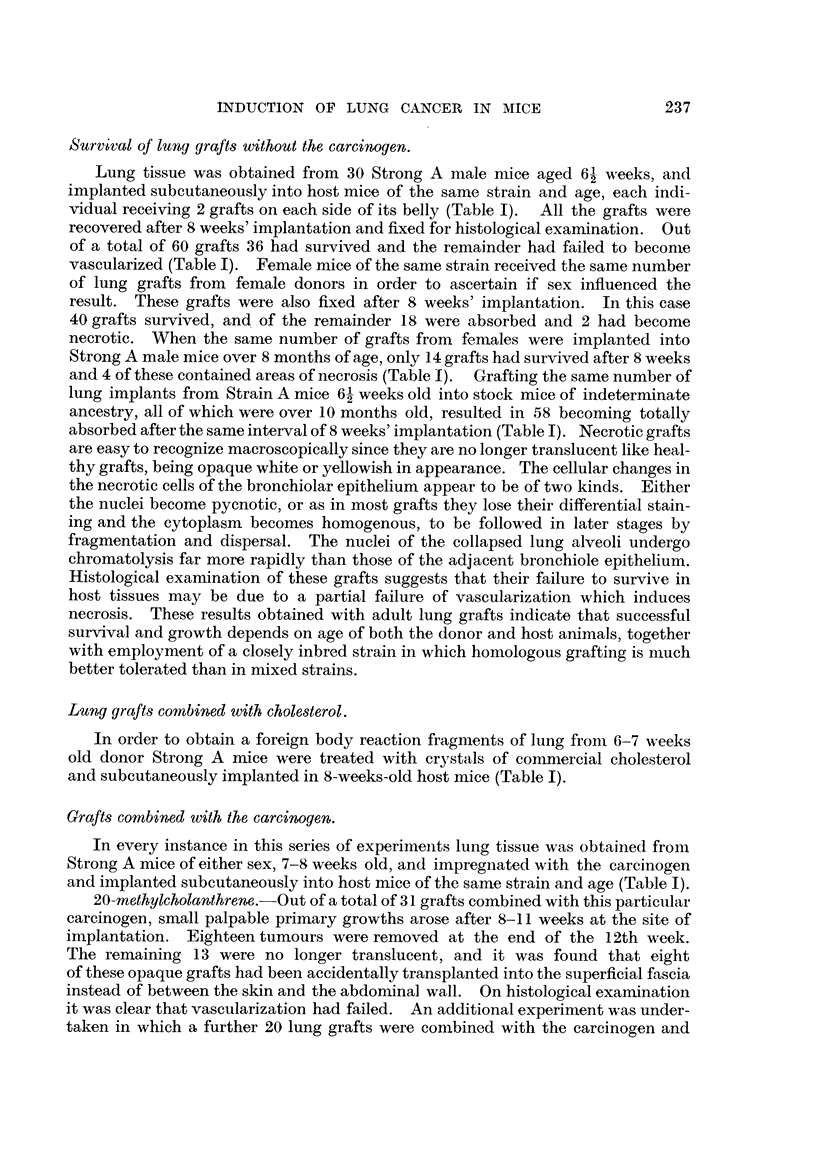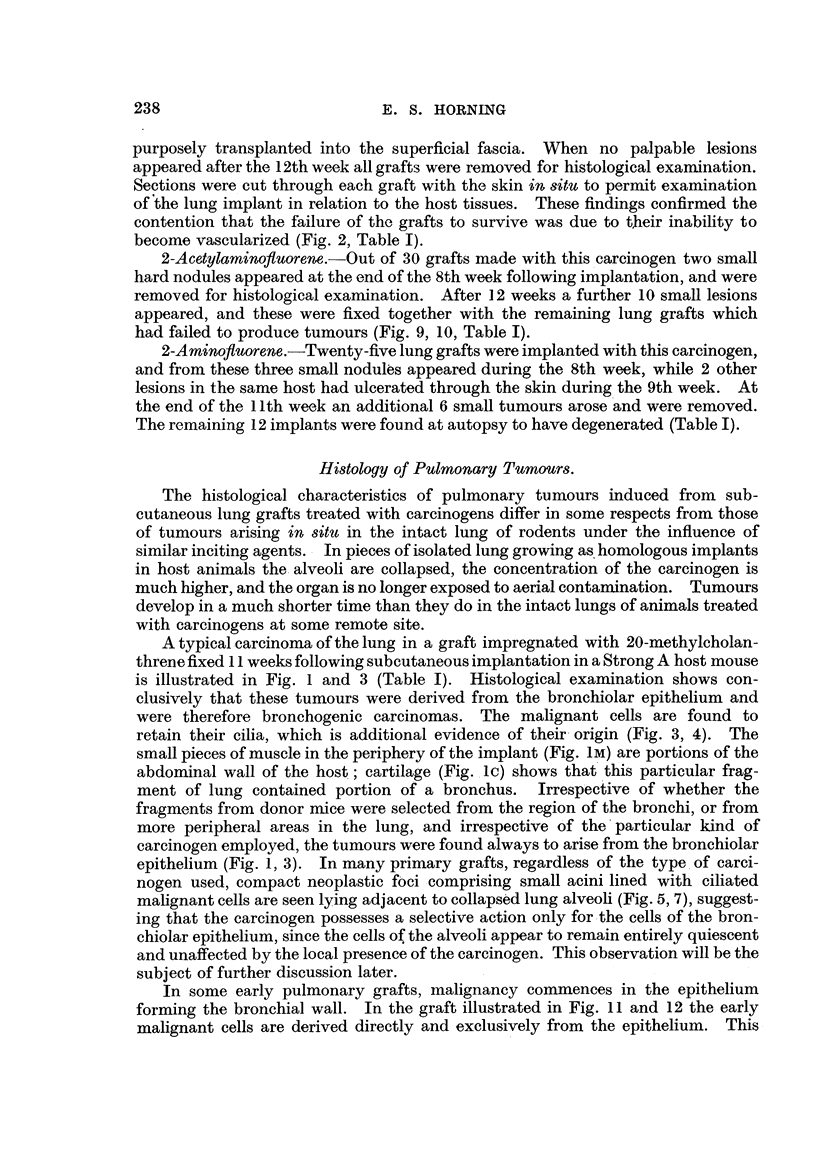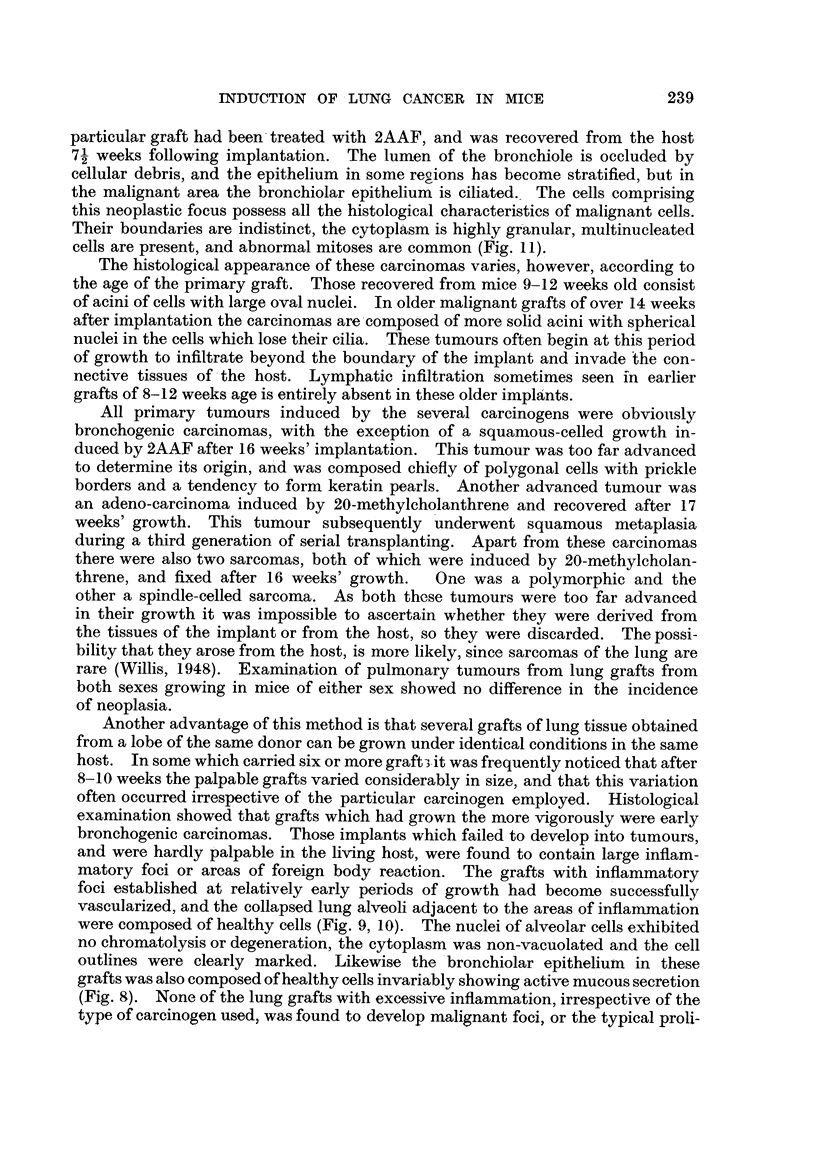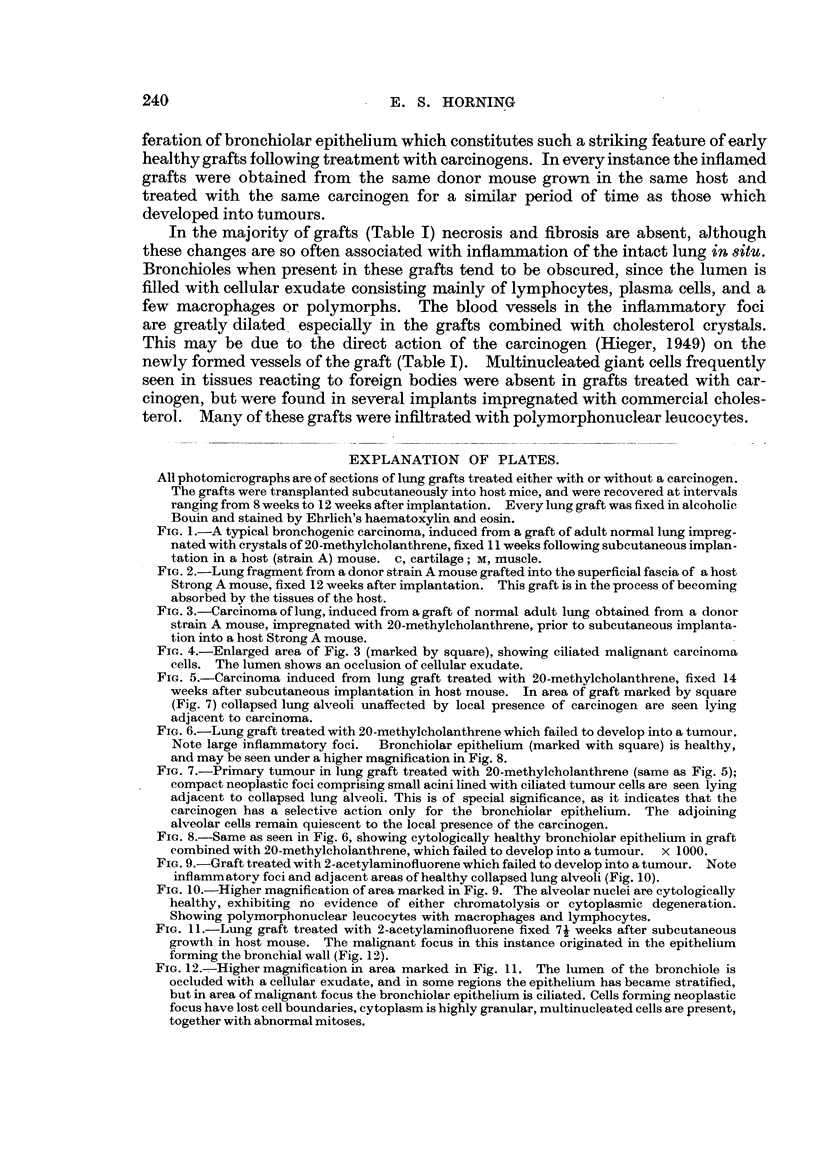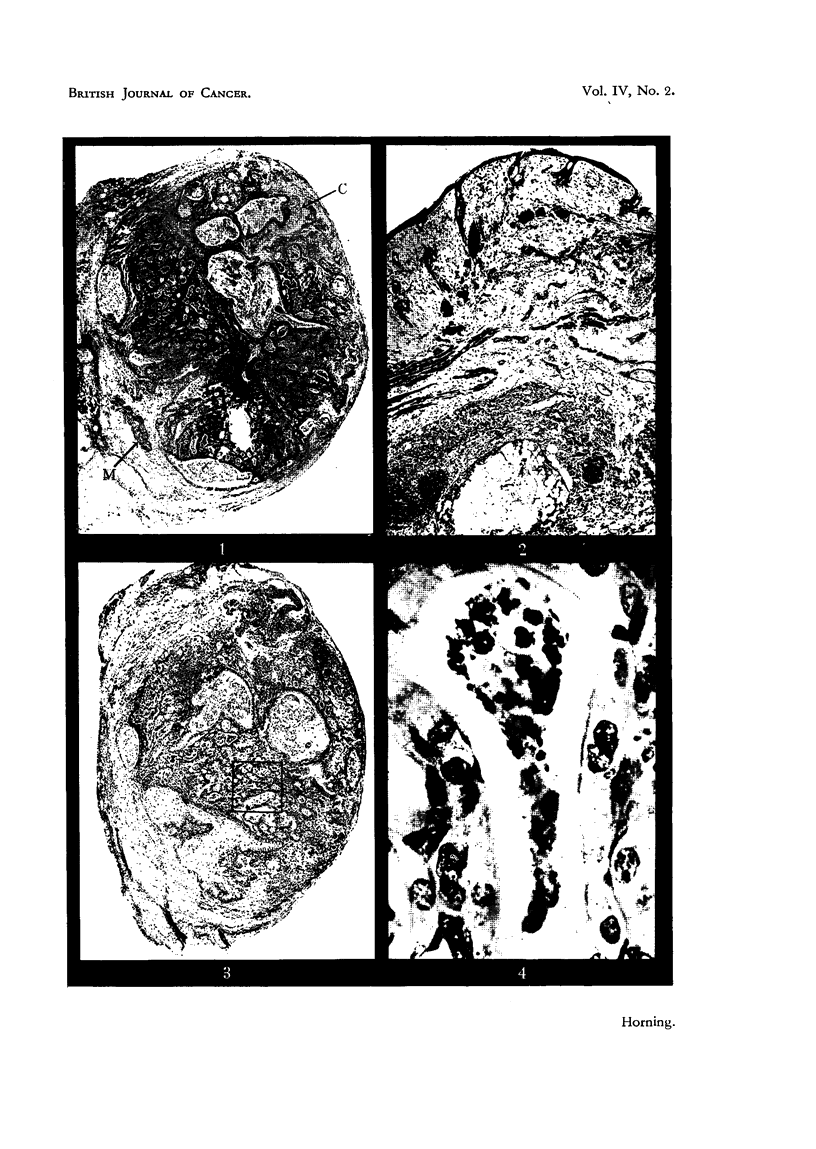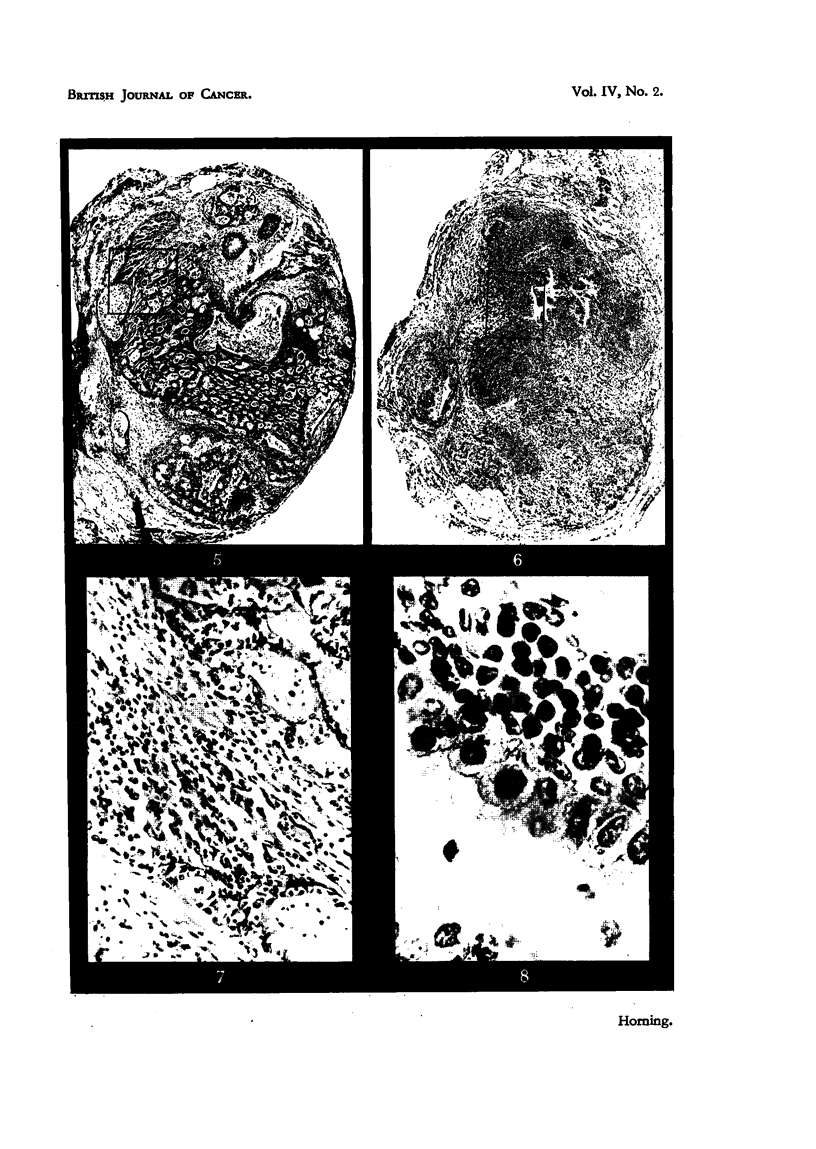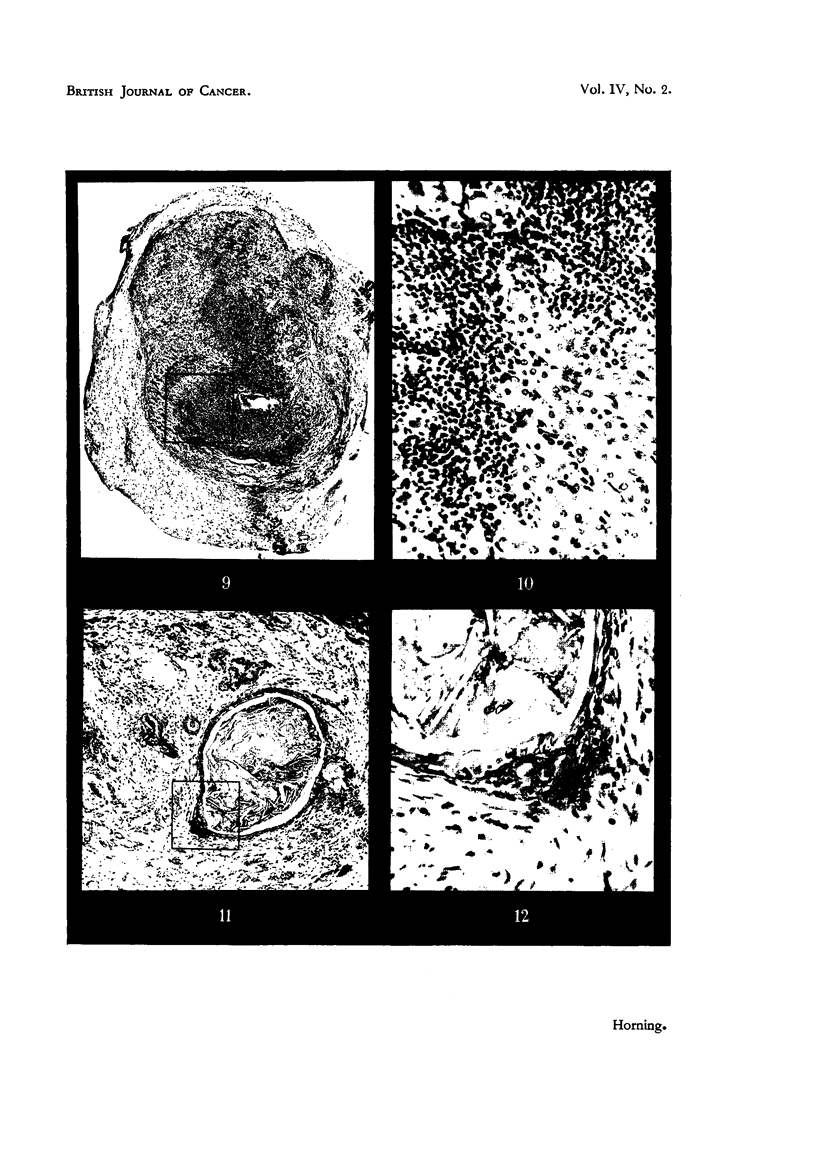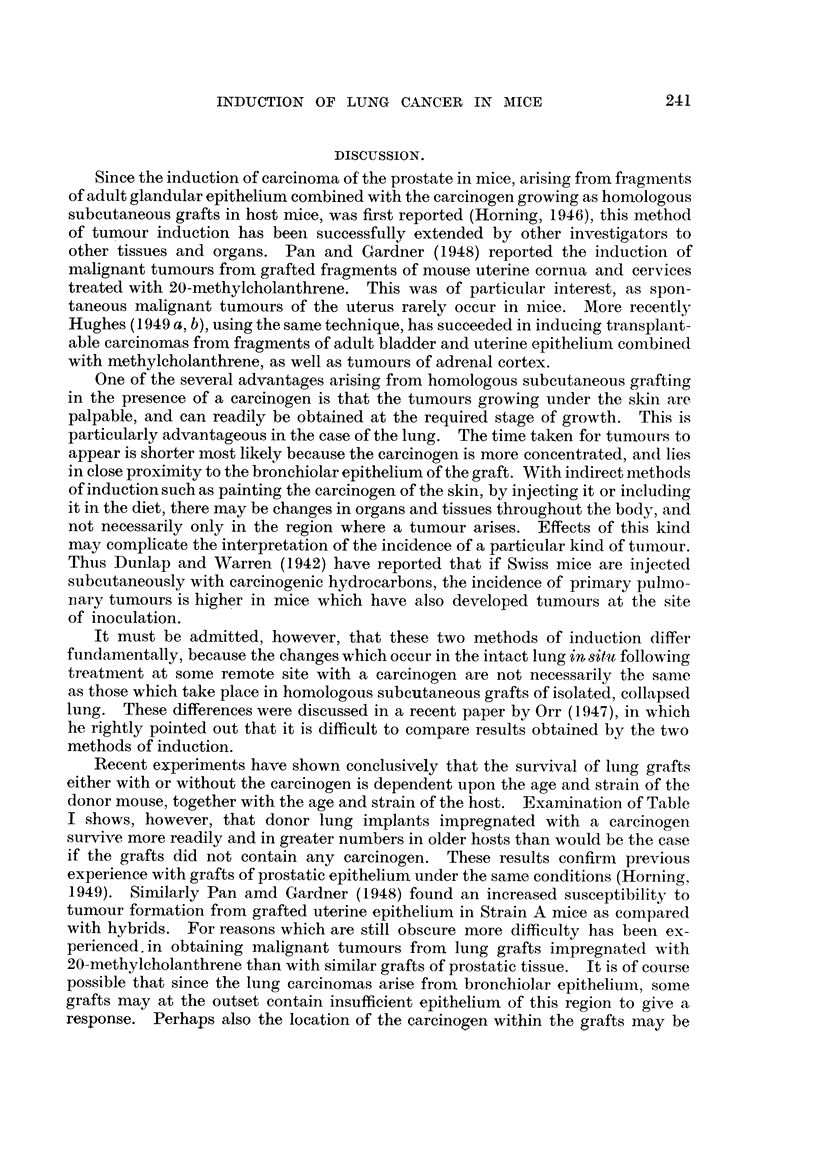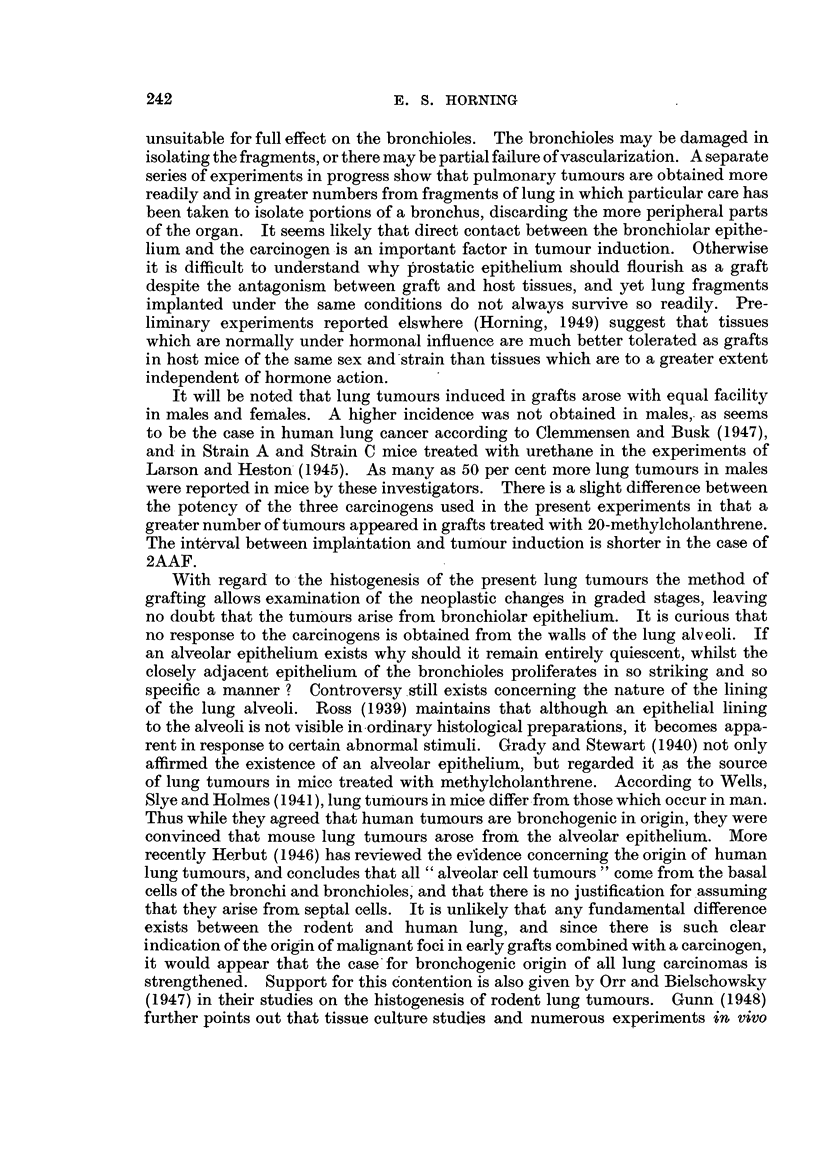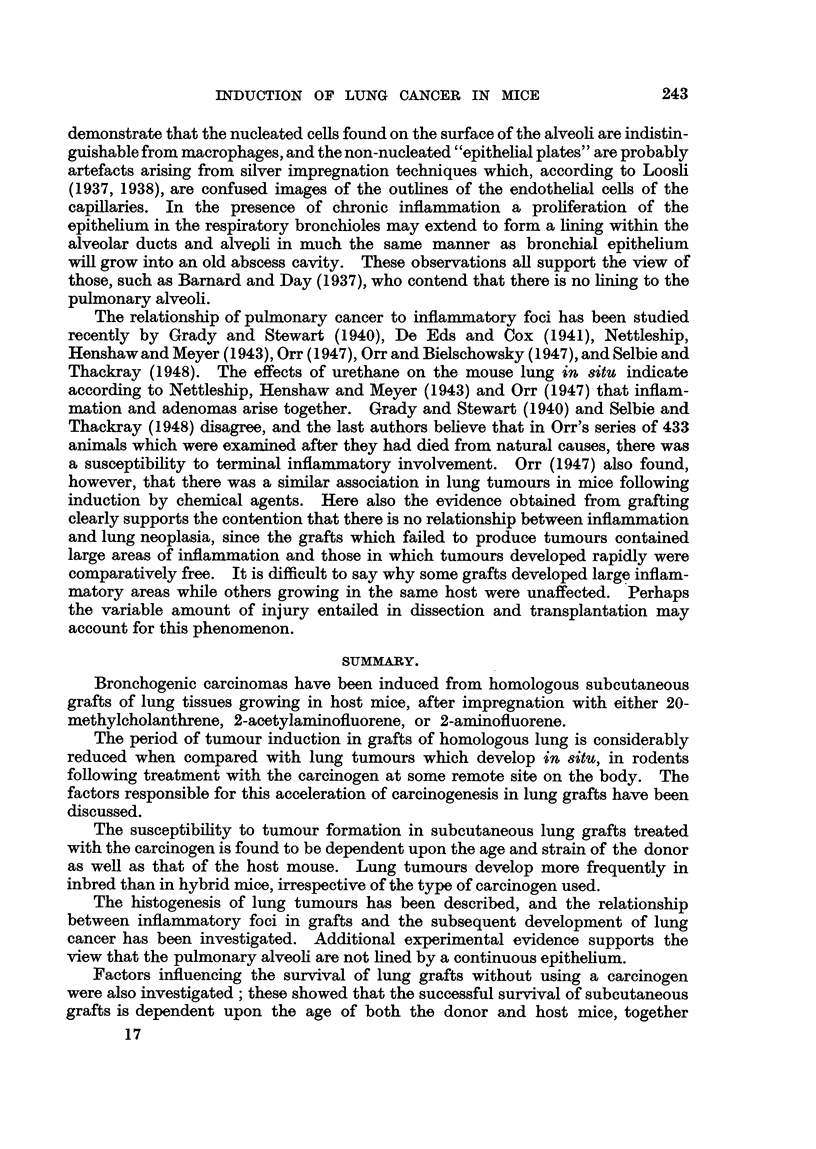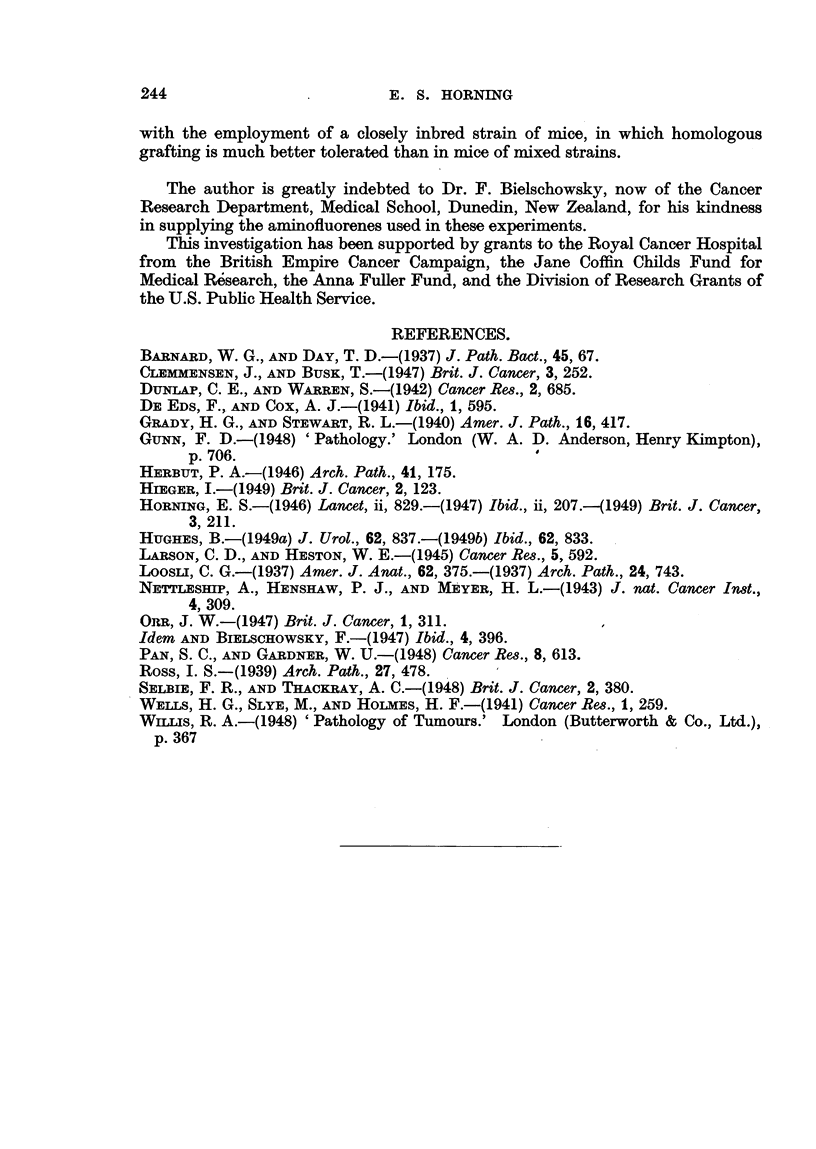# Studies on the Induction of Lung Cancer in Mice

**DOI:** 10.1038/bjc.1950.23

**Published:** 1950-06

**Authors:** E. S. Horning

## Abstract

**Images:**


### STUDIES ON THE INDUCTION OF LUNG CANCER IN MICE.

E. S. HORNING.

*From the Chester Beatty Research Institute, The Royal Cancer Hospital, London, S.W.3.*

Received for publication April 13, 1950.

LUNG tissue is unsuitable for direct injection of carcinogens, consequently pulmonary tumours in laboratory animals have hitherto been induced by applying the carcinogen at some remote site, by injecting it subcutaneously, intratracheally, intravascularly, intraperitoneally, or by including it in the diet. These indirect methods are often laborious, and tumour induction may take a considerable time.

Recently the author (Horning, 1947) reported the induction of pulmonary tumours after relatively short periods of treatment by the direct application of carcinogens to adult lung tissue growing as subcutaneous homologous grafts in host animals. Further experiments have shown that success with this technique depends upon several factors, and the object of this paper is to describe additional observations.

### TECHNIQUE.

The method of tumour induction consists in isolating small strips of lung from 6–12 weeks Strain A mice, impregnating the grafts with crystals of a carcinogen (2-acetylaminofluorene, 2-aminofluorene, or 20-methylcholanthrene) prior to implanting them subcutaneously into host mice of similar age and strain in a manner which has been described elswhere (Horning, 1947). Usually 4 subcutaneous grafts were made on each side of the abdomen of a single host mouse in order to have the implants growing under identical conditions. Small palpable tumours were obtained at intervals ranging from 6–12 weeks following grafting, but in order to examine the differences between the responses to individual carcinogens, grafts were fixed before tumours developed at intervals ranging from 1–5 weeks after implantation.

Lung fragments from 6½ weeks old Strain A mice were grafted, in some instances without the carcinogen, into host mice of similar age, and also into mice of 15 months age which were selected from stock mice of undetermined ancestry. This was done to find out if the survival of homologous grafts was dependent on employing a closely inbred strain, or whether it might be influenced by the age of the host.

In another series of experiments a number of lung grafts were treated with 20-methylcholanthrene, half being implanted into the superficial fascia, and the remainder placed as usual between the skin and the abdominal wall. The host mice were again of the same age and strain, and it was thought that some information might be obtained as to whether rapid vascularization played any role in successful survival of the grafts.

TABLE I.

| Donor mouse. | | | Number of mice. | Host mouse. | | | Carcinogen. | Grafts. | | | Number of tumours. | Histology of grafts. |
|---|---|---|---|---|---|---|---|---|---|---|---|---|
| Strain. | Sex. | Age (weeks). | | Strain. | Sex. | Age (weeks). | | Number of grafts. | Period of implantation (weeks). | Number of survivals. | | |
| A | ♂ | 6½ | 30 | A | ♂ | 6½ | None | 60 | 8 | 36 | None | 36 grafts owing to failure of vascularization in host tissues became necrotic; remainder were totally absorbed. |
| A | ♀ | 6 | 30 | A | ♀ | 6 | ,, | 60 | 8 | 40 | ,, | 18 grafts became absorbed in tissues of host mice. Two others were necrotic. |
| A | ♀ | 32 | 30 | A | ♂ | 32 | ,, | 59 | 8 | 14 | ,, | 14 grafts survived; 4 contained areas of necrosis. |
| A | ♂ | 6½ | 30 | Hybrid | ♂ | 40 | ,, | 60 | 8 | 2 | ,, | 58 grafts became totally absorbed in tissues of host mice. |
| A | ♂ | 6½ | 15 | A | ♂ | 8 | Commercial cholesterol | 15 | 9 | 8 | ,, | Foreign body reaction. Many grafts contained multi-nucleated giant cells. |
| A | ♂♀ | 7½ | 16 | A | ♂♀ | 7½ | 20-methyl-cholanthrene | 31 | 8–11 | 18 | 18 | Bronchogenic carcinoma. Remaining 13 grafts either became absorbed or were necrotic. |
| A | ♂ | 12 | 20 | A | ♂ | 11 | 20-methyl-cholanthrene | 20 | 12 implanted into superficial fascia | None | None | All grafts failed to become vascularized and were in process of absorption. |
| A | ♂ | 8 | 15 | A | ♂ | 10 | 2-acetyl amino-fluorene | 30 | 8–12 | 16 | 12 | 12 adenomas; Remaining grafts were either necrotic or absorbed. |
| A | ♂ | 8½ | 16 | A | ♂ | 9 | 2-amino-fluorene | 25 | 8–13 | 20 | 13 | 11 adenomas, 2 early bronchogenic carcinomas. |
| A | ♂♀ | 7½ | 15 | A | ♂♀ | 8 | 20-methyl-cholanthrene | 30 | 1–5 | 28 | None | 2 grafts necrotic; remainder showed bronchiolar proliferation. |
| A | ♂♀ | 8 | 16 | A | ♂♀ | 7½ | 2-acetyl-amino-fluorene | 25 | 1–5 | 22 | ,, | 3 grafts were absorbed. |
| A | ♂♀ | 7½ | 15 | A | ♂♀ | 8 | 2-amino-fluorene | 25 | 1–5 | 21 | ,, | 2 grafts had become necrotic, 1 had been absorbed. |

*Survival of lung grafts without the carcinogen.*

Lung tissue was obtained from 30 Strong A male mice aged 6½ weeks, and implanted subcutaneously into host mice of the same strain and age, each individual receiving 2 grafts on each side of its belly (Table I). All the grafts were recovered after 8 weeks' implantation and fixed for histological examination. Out of a total of 60 grafts 36 had survived and the remainder had failed to become vascularized (Table I). Female mice of the same strain received the same number of lung grafts from female donors in order to ascertain if sex influenced the result. These grafts were also fixed after 8 weeks' implantation. In this case 40 grafts survived, and of the remainder 18 were absorbed and 2 had become necrotic. When the same number of grafts from females were implanted into Strong A male mice over 8 months of age, only 14 grafts had survived after 8 weeks and 4 of these contained areas of necrosis (Table I). Grafting the same number of lung implants from Strain A mice 6½ weeks old into stock mice of indeterminate ancestry, all of which were over 10 months old, resulted in 58 becoming totally absorbed after the same interval of 8 weeks' implantation (Table I). Necrotic grafts are easy to recognize macroscopically since they are no longer translucent like healthy grafts, being opaque white or yellowish in appearance. The cellular changes in the necrotic cells of the bronchiolar epithelium appear to be of two kinds. Either the nuclei become pycnotic, or as in most grafts they lose their differential staining and the cytoplasm becomes homogenous, to be followed in later stages by fragmentation and dispersal. The nuclei of the collapsed lung alveoli undergo chromatolysis far more rapidly than those of the adjacent bronchiole epithelium. Histological examination of these grafts suggests that their failure to survive in host tissues may be due to a partial failure of vascularization which induces necrosis. These results obtained with adult lung grafts indicate that successful survival and growth depends on age of both the donor and host animals, together with employment of a closely inbred strain in which homologous grafting is much better tolerated than in mixed strains.

*Lung grafts combined with cholesterol.*

In order to obtain a foreign body reaction fragments of lung from 6–7 weeks old donor Strong A mice were treated with crystals of commercial cholesterol and subcutaneously implanted in 8-weeks-old host mice (Table I).

*Grafts combined with the carcinogen.*

In every instance in this series of experiments lung tissue was obtained from Strong A mice of either sex, 7–8 weeks old, and impregnated with the carcinogen and implanted subcutaneously into host mice of the same strain and age (Table I).

20-*methylcholanthrene*.—Out of a total of 31 grafts combined with this particular carcinogen, small palpable primary growths arose after 8–11 weeks at the site of implantation. Eighteen tumours were removed at the end of the 12th week. The remaining 13 were no longer translucent, and it was found that eight of these opaque grafts had been accidentally transplanted into the superficial fascia instead of between the skin and the abdominal wall. On histological examination it was clear that vascularization had failed. An additional experiment was undertaken in which a further 20 lung grafts were combined with the carcinogen and purposely transplanted into the superficial fascia. When no palpable lesions appeared after the 12th week all grafts were removed for histological examination. Sections were cut through each graft with the skin *in situ* to permit examination of the lung implant in relation to the host tissues. These findings confirmed the contention that the failure of the grafts to survive was due to their inability to become vascularized (Fig. 2, Table I).

2-*Acetylaminofluorene.*—Out of 30 grafts made with this carcinogen two small hard nodules appeared at the end of the 8th week following implantation, and were removed for histological examination. After 12 weeks a further 10 small lesions appeared, and these were fixed together with the remaining lung grafts which had failed to produce tumours (Fig. 9, 10, Table I).

2-*Aminofluorene.*—Twenty-five lung grafts were implanted with this carcinogen, and from these three small nodules appeared during the 8th week, while 2 other lesions in the same host had ulcerated through the skin during the 9th week. At the end of the 11th week an additional 6 small tumours arose and were removed. The remaining 12 implants were found at autopsy to have degenerated (Table I).

*Histology of Pulmonary Tumours.*

The histological characteristics of pulmonary tumours induced from subcutaneous lung grafts treated with carcinogens differ in some respects from those of tumours arising *in situ* in the intact lung of rodents under the influence of similar inciting agents. In pieces of isolated lung growing as homologous implants in host animals the alveoli are collapsed, the concentration of the carcinogen is much higher, and the organ is no longer exposed to aerial contamination. Tumours develop in a much shorter time than they do in the intact lungs of animals treated with carcinogens at some remote site.

A typical carcinoma of the lung in a graft impregnated with 20-methylcholanthrene fixed 11 weeks following subcutaneous implantation in a Strong A host mouse is illustrated in Fig. 1 and 3 (Table I). Histological examination shows conclusively that these tumours were derived from the bronchiolar epithelium and were therefore bronchogenic carcinomas. The malignant cells are found to retain their cilia, which is additional evidence of their origin (Fig. 3, 4). The small pieces of muscle in the periphery of the implant (Fig. 1M) are portions of the abdominal wall of the host; cartilage (Fig. 1C) shows that this particular fragment of lung contained portion of a bronchus. Irrespective of whether the fragments from donor mice were selected from the region of the bronchi, or from more peripheral areas in the lung, and irrespective of the particular kind of carcinogen employed, the tumours were found always to arise from the bronchiolar epithelium (Fig. 1, 3). In many primary grafts, regardless of the type of carcinogen used, compact neoplastic foci comprising small acini lined with ciliated malignant cells are seen lying adjacent to collapsed lung alveoli (Fig. 5, 7), suggesting that the carcinogen possesses a selective action only for the cells of the bronchiolar epithelium, since the cells of the alveoli appear to remain entirely quiescent and unaffected by the local presence of the carcinogen. This observation will be the subject of further discussion later.

In some early pulmonary grafts, malignancy commences in the epithelium forming the bronchial wall. In the graft illustrated in Fig. 11 and 12 the early malignant cells are derived directly and exclusively from the epithelium. This particular graft had been treated with 2AAF, and was recovered from the host $7\frac{1}{2}$ weeks following implantation. The lumen of the bronchiole is occluded by cellular debris, and the epithelium in some regions has become stratified, but in the malignant area the bronchiolar epithelium is ciliated. The cells comprising this neoplastic focus possess all the histological characteristics of malignant cells. Their boundaries are indistinct, the cytoplasm is highly granular, multinucleated cells are present, and abnormal mitoses are common (Fig. 11).

The histological appearance of these carcinomas varies, however, according to the age of the primary graft. Those recovered from mice 9–12 weeks old consist of acini of cells with large oval nuclei. In older malignant grafts of over 14 weeks after implantation the carcinomas are composed of more solid acini with spherical nuclei in the cells which lose their cilia. These tumours often begin at this period of growth to infiltrate beyond the boundary of the implant and invade the connective tissues of the host. Lymphatic infiltration sometimes seen in earlier grafts of 8–12 weeks age is entirely absent in these older implants.

All primary tumours induced by the several carcinogens were obviously bronchogenic carcinomas, with the exception of a squamous-celled growth induced by 2AAF after 16 weeks' implantation. This tumour was too far advanced to determine its origin, and was composed chiefly of polygonal cells with prickle borders and a tendency to form keratin pearls. Another advanced tumour was an adeno-carcinoma induced by 20-methylcholanthrene and recovered after 17 weeks' growth. This tumour subsequently underwent squamous metaplasia during a third generation of serial transplanting. Apart from these carcinomas there were also two sarcomas, both of which were induced by 20-methylcholanthrene, and fixed after 16 weeks' growth. One was a polymorphic and the other a spindle-celled sarcoma. As both these tumours were too far advanced in their growth it was impossible to ascertain whether they were derived from the tissues of the implant or from the host, so they were discarded. The possibility that they arose from the host, is more likely, since sarcomas of the lung are rare (Willis, 1948). Examination of pulmonary tumours from lung grafts from both sexes growing in mice of either sex showed no difference in the incidence of neoplasia.

Another advantage of this method is that several grafts of lung tissue obtained from a lobe of the same donor can be grown under identical conditions in the same host. In some which carried six or more grafts it was frequently noticed that after 8–10 weeks the palpable grafts varied considerably in size, and that this variation often occurred irrespective of the particular carcinogen employed. Histological examination showed that grafts which had grown the more vigorously were early bronchogenic carcinomas. Those implants which failed to develop into tumours, and were hardly palpable in the living host, were found to contain large inflammatory foci or areas of foreign body reaction. The grafts with inflammatory foci established at relatively early periods of growth had become successfully vascularized, and the collapsed lung alveoli adjacent to the areas of inflammation were composed of healthy cells (Fig. 9, 10). The nuclei of alveolar cells exhibited no chromatolysis or degeneration, the cytoplasm was non-vacuolated and the cell outlines were clearly marked. Likewise the bronchiolar epithelium in these grafts was also composed of healthy cells invariably showing active mucous secretion (Fig. 8). None of the lung grafts with excessive inflammation, irrespective of the type of carcinogen used, was found to develop malignant foci, or the typical proliferation of bronchiolar epithelium which constitutes such a striking feature of early healthy grafts following treatment with carcinogens. In every instance the inflamed grafts were obtained from the same donor mouse grown in the same host and treated with the same carcinogen for a similar period of time as those which developed into tumours.

In the majority of grafts (Table I) necrosis and fibrosis are absent, although these changes are so often associated with inflammation of the intact lung *in situ*. Bronchioles when present in these grafts tend to be obscured, since the lumen is filled with cellular exudate consisting mainly of lymphocytes, plasma cells, and a few macrophages or polymorphs. The blood vessels in the inflammatory foci are greatly dilated especially in the grafts combined with cholesterol crystals. This may be due to the direct action of the carcinogen (Hieger, 1949) on the newly formed vessels of the graft (Table I). Multinucleated giant cells frequently seen in tissues reacting to foreign bodies were absent in grafts treated with carcinogen, but were found in several implants impregnated with commercial cholesterol. Many of these grafts were infiltrated with polymorphonuclear leucocytes.

### EXPLANATION OF PLATES.

All photomicrographs are of sections of lung grafts treated either with or without a carcinogen. The grafts were transplanted subcutaneously into host mice, and were recovered at intervals ranging from 8 weeks to 12 weeks after implantation. Every lung graft was fixed in alcoholic Bouin and stained by Ehrlich's haematoxylin and eosin.

FIG. 1.—A typical bronchogenic carcinoma, induced from a graft of adult normal lung impregnated with crystals of 20-methylcholanthrene, fixed 11 weeks following subcutaneous implantation in a host (strain A) mouse. C, cartilage ; M, muscle.

FIG. 2.—Lung fragment from a donor strain A mouse grafted into the superficial fascia of a host Strong A mouse, fixed 12 weeks after implantation. This graft is in the process of becoming absorbed by the tissues of the host.

FIG. 3.—Carcinoma of lung, induced from a graft of normal adult lung obtained from a donor strain A mouse, impregnated with 20-methylcholanthrene, prior to subcutaneous implantation into a host Strong A mouse.

FIG. 4.—Enlarged area of Fig. 3 (marked by square), showing ciliated malignant carcinoma cells. The lumen shows an occlusion of cellular exudate.

FIG. 5.—Carcinoma induced from lung graft treated with 20-methylcholanthrene, fixed 14 weeks after subcutaneous implantation in host mouse. In area of graft marked by square (Fig. 7) collapsed lung alveoli unaffected by local presence of carcinogen are seen lying adjacent to carcinoma.

FIG. 6.—Lung graft treated with 20-methylcholanthrene which failed to develop into a tumour. Note large inflammatory foci. Bronchiolar epithelium (marked with square) is healthy, and may be seen under a higher magnification in Fig. 8.

FIG. 7.—Primary tumour in lung graft treated with 20-methylcholanthrene (same as Fig. 5); compact neoplastic foci comprising small acini lined with ciliated tumour cells are seen lying adjacent to collapsed lung alveoli. This is of special significance, as it indicates that the carcinogen has a selective action only for the bronchiolar epithelium. The adjoining alveolar cells remain quiescent to the local presence of the carcinogen.

FIG. 8.—Same as seen in Fig. 6, showing cytologically healthy bronchiolar epithelium in graft combined with 20-methylcholanthrene, which failed to develop into a tumour. × 1000.

FIG. 9.—Graft treated with 2-acetylaminofluorene which failed to develop into a tumour. Note inflammatory foci and adjacent areas of healthy collapsed lung alveoli (Fig. 10).

FIG. 10.—Higher magnification of area marked in Fig. 9. The alveolar nuclei are cytologically healthy, exhibiting no evidence of either chromatolysis or cytoplasmic degeneration. Showing polymorphonuclear leucocytes with macrophages and lymphocytes.

FIG. 11.—Lung graft treated with 2-acetylaminofluorene fixed 7½ weeks after subcutaneous growth in host mouse. The malignant focus in this instance originated in the epithelium forming the bronchial wall (Fig. 12).

FIG. 12.—Higher magnification in area marked in Fig. 11. The lumen of the bronchiole is occluded with a cellular exudate, and in some regions the epithelium has became stratified, but in area of malignant focus the bronchiolar epithelium is ciliated. Cells forming neoplastic focus have lost cell boundaries, cytoplasm is highly granular, multinucleated cells are present, together with abnormal mitoses.

C
M
1
2
3
4

5

6

7

8

Horning.

9

10

11

12

Horning.

### DISCUSSION.

Since the induction of carcinoma of the prostate in mice, arising from fragments of adult glandular epithelium combined with the carcinogen growing as homologous subcutaneous grafts in host mice, was first reported (Horning, 1946), this method of tumour induction has been successfully extended by other investigators to other tissues and organs. Pan and Gardner (1948) reported the induction of malignant tumours from grafted fragments of mouse uterine cornua and cervices treated with 20-methylcholanthrene. This was of particular interest, as spontaneous malignant tumours of the uterus rarely occur in mice. More recently Hughes (1949 *a*, *b*), using the same technique, has succeeded in inducing transplantable carcinomas from fragments of adult bladder and uterine epithelium combined with methylcholanthrene, as well as tumours of adrenal cortex.

One of the several advantages arising from homologous subcutaneous grafting in the presence of a carcinogen is that the tumours growing under the skin are palpable, and can readily be obtained at the required stage of growth. This is particularly advantageous in the case of the lung. The time taken for tumours to appear is shorter most likely because the carcinogen is more concentrated, and lies in close proximity to the bronchiolar epithelium of the graft. With indirect methods of induction such as painting the carcinogen of the skin, by injecting it or including it in the diet, there may be changes in organs and tissues throughout the body, and not necessarily only in the region where a tumour arises. Effects of this kind may complicate the interpretation of the incidence of a particular kind of tumour. Thus Dunlap and Warren (1942) have reported that if Swiss mice are injected subcutaneously with carcinogenic hydrocarbons, the incidence of primary pulmonary tumours is higher in mice which have also developed tumours at the site of inoculation.

It must be admitted, however, that these two methods of induction differ fundamentally, because the changes which occur in the intact lung *in situ* following treatment at some remote site with a carcinogen are not necessarily the same as those which take place in homologous subcutaneous grafts of isolated, collapsed lung. These differences were discussed in a recent paper by Orr (1947), in which he rightly pointed out that it is difficult to compare results obtained by the two methods of induction.

Recent experiments have shown conclusively that the survival of lung grafts either with or without the carcinogen is dependent upon the age and strain of the donor mouse, together with the age and strain of the host. Examination of Table I shows, however, that donor lung implants impregnated with a carcinogen survive more readily and in greater numbers in older hosts than would be the case if the grafts did not contain any carcinogen. These results confirm previous experience with grafts of prostatic epithelium under the same conditions (Horning, 1949). Similarly Pan amd Gardner (1948) found an increased susceptibility to tumour formation from grafted uterine epithelium in Strain A mice as compared with hybrids. For reasons which are still obscure more difficulty has been experienced.in obtaining malignant tumours from lung grafts impregnated with 20-methylcholanthrene than with similar grafts of prostatic tissue. It is of course possible that since the lung carcinomas arise from bronchiolar epithelium, some grafts may at the outset contain insufficient epithelium of this region to give a response. Perhaps also the location of the carcinogen within the grafts may be unsuitable for full effect on the bronchioles. The bronchioles may be damaged in isolating the fragments, or there may be partial failure of vascularization. A separate series of experiments in progress show that pulmonary tumours are obtained more readily and in greater numbers from fragments of lung in which particular care has been taken to isolate portions of a bronchus, discarding the more peripheral parts of the organ. It seems likely that direct contact between the bronchiolar epithelium and the carcinogen is an important factor in tumour induction. Otherwise it is difficult to understand why prostatic epithelium should flourish as a graft despite the antagonism between graft and host tissues, and yet lung fragments implanted under the same conditions do not always survive so readily. Preliminary experiments reported elswhere (Horning, 1949) suggest that tissues which are normally under hormonal influence are much better tolerated as grafts in host mice of the same sex and strain than tissues which are to a greater extent independent of hormone action.

It will be noted that lung tumours induced in grafts arose with equal facility in males and females. A higher incidence was not obtained in males, as seems to be the case in human lung cancer according to Clemmensen and Busk (1947), and in Strain A and Strain C mice treated with urethane in the experiments of Larson and Heston (1945). As many as 50 per cent more lung tumours in males were reported in mice by these investigators. There is a slight difference between the potency of the three carcinogens used in the present experiments in that a greater number of tumours appeared in grafts treated with 20-methylcholanthrene. The interval between implantation and tumour induction is shorter in the case of 2AAF.

With regard to the histogenesis of the present lung tumours the method of grafting allows examination of the neoplastic changes in graded stages, leaving no doubt that the tumours arise from bronchiolar epithelium. It is curious that no response to the carcinogens is obtained from the walls of the lung alveoli. If an alveolar epithelium exists why should it remain entirely quiescent, whilst the closely adjacent epithelium of the bronchioles proliferates in so striking and so specific a manner ? Controversy still exists concerning the nature of the lining of the lung alveoli. Ross (1939) maintains that although an epithelial lining to the alveoli is not visible in ordinary histological preparations, it becomes apparent in response to certain abnormal stimuli. Grady and Stewart (1940) not only affirmed the existence of an alveolar epithelium, but regarded it as the source of lung tumours in mice treated with methylcholanthrene. According to Wells, Slye and Holmes (1941), lung tumours in mice differ from those which occur in man. Thus while they agreed that human tumours are bronchogenic in origin, they were convinced that mouse lung tumours arose from the alveolar epithelium. More recently Herbut (1946) has reviewed the evidence concerning the origin of human lung tumours, and concludes that all " alveolar cell tumours " come from the basal cells of the bronchi and bronchioles, and that there is no justification for assuming that they arise from septal cells. It is unlikely that any fundamental difference exists between the rodent and human lung, and since there is such clear indication of the origin of malignant foci in early grafts combined with a carcinogen, it would appear that the case for bronchogenic origin of all lung carcinomas is strengthened. Support for this contention is also given by Orr and Bielschowsky (1947) in their studies on the histogenesis of rodent lung tumours. Gunn (1948) further points out that tissue culture studies and numerous experiments *in vivo*

demonstrate that the nucleated cells found on the surface of the alveoli are indistinguishable from macrophages, and the non-nucleated "epithelial plates" are probably artefacts arising from silver impregnation techniques which, according to Loosli (1937, 1938), are confused images of the outlines of the endothelial cells of the capillaries. In the presence of chronic inflammation a proliferation of the epithelium in the respiratory bronchioles may extend to form a lining within the alveolar ducts and alveoli in much the same manner as bronchial epithelium will grow into an old abscess cavity. These observations all support the view of those, such as Barnard and Day (1937), who contend that there is no lining to the pulmonary alveoli.

The relationship of pulmonary cancer to inflammatory foci has been studied recently by Grady and Stewart (1940), De Eds and Cox (1941), Nettleship, Henshaw and Meyer (1943), Orr (1947), Orr and Bielschowsky (1947), and Selbie and Thackray (1948). The effects of urethane on the mouse lung *in situ* indicate according to Nettleship, Henshaw and Meyer (1943) and Orr (1947) that inflammation and adenomas arise together. Grady and Stewart (1940) and Selbie and Thackray (1948) disagree, and the last authors believe that in Orr's series of 433 animals which were examined after they had died from natural causes, there was a susceptibility to terminal inflammatory involvement. Orr (1947) also found, however, that there was a similar association in lung tumours in mice following induction by chemical agents. Here also the evidence obtained from grafting clearly supports the contention that there is no relationship between inflammation and lung neoplasia, since the grafts which failed to produce tumours contained large areas of inflammation and those in which tumours developed rapidly were comparatively free. It is difficult to say why some grafts developed large inflammatory areas while others growing in the same host were unaffected. Perhaps the variable amount of injury entailed in dissection and transplantation may account for this phenomenon.

### SUMMARY.

Bronchogenic carcinomas have been induced from homologous subcutaneous grafts of lung tissues growing in host mice, after impregnation with either 20-methylcholanthrene, 2-acetylaminofluorene, or 2-aminofluorene.

The period of tumour induction in grafts of homologous lung is considerably reduced when compared with lung tumours which develop *in situ*, in rodents following treatment with the carcinogen at some remote site on the body. The factors responsible for this acceleration of carcinogenesis in lung grafts have been discussed.

The susceptibility to tumour formation in subcutaneous lung grafts treated with the carcinogen is found to be dependent upon the age and strain of the donor as well as that of the host mouse. Lung tumours develop more frequently in inbred than in hybrid mice, irrespective of the type of carcinogen used.

The histogenesis of lung tumours has been described, and the relationship between inflammatory foci in grafts and the subsequent development of lung cancer has been investigated. Additional experimental evidence supports the view that the pulmonary alveoli are not lined by a continuous epithelium.

Factors influencing the survival of lung grafts without using a carcinogen were also investigated ; these showed that the successful survival of subcutaneous grafts is dependent upon the age of both the donor and host mice, together with the employment of a closely inbred strain of mice, in which homologous grafting is much better tolerated than in mice of mixed strains.

The author is greatly indebted to Dr. F. Bielschowsky, now of the Cancer Research Department, Medical School, Dunedin, New Zealand, for his kindness in supplying the aminofluorenes used in these experiments.

This investigation has been supported by grants to the Royal Cancer Hospital from the British Empire Cancer Campaign, the Jane Coffin Childs Fund for Medical Research, the Anna Fuller Fund, and the Division of Research Grants of the U.S. Public Health Service.

### REFERENCES.

BARNARD, W. G., AND DAY, T. D.—(1937) *J. Path. Bact.*, **45**, 67.

CLEMMENSEN, J., AND BUSK, T.—(1947) *Brit. J. Cancer*, **3**, 252.

DUNLAP, C. E., AND WARREN, S.—(1942) *Cancer Res.*, **2**, 685.

DE EDS, F., AND COX, A. J.—(1941) *Ibid.*, **1**, 595.

GRADY, H. G., AND STEWART, R. L.—(1940) *Amer. J. Path.*, **16**, 417.

GUNN, F. D.—(1948) 'Pathology.' London (W. A. D. Anderson, Henry Kimpton), p. 706.

HERBUT, P. A.—(1946) *Arch. Path.*, **41**, 175.

HIEGER, I.—(1949) *Brit. J. Cancer*, **2**, 123.

HORNING, E. S.—(1946) *Lancet*, ii, 829.—(1947) *Ibid.*, ii, 207.—(1949) *Brit. J. Cancer*, **3**, 211.

HUGHES, B.—(1949*a*) *J. Urol.*, **62**, 837.—(1949*b*) *Ibid.*, **62**, 833.

LARSON, C. D., AND HESTON, W. E.—(1945) *Cancer Res.*, **5**, 592.

LOOSLI, C. G.—(1937) *Amer. J. Anat.*, **62**, 375.—(1937) *Arch. Path.*, **24**, 743.

NETTLESHIP, A., HENSHAW, P. J., AND MEYER, H. L.—(1943) *J. nat. Cancer Inst.*, **4**, 309.

ORR, J. W.—(1947) *Brit. J. Cancer*, **1**, 311.

*Idem* AND BIELSCHOWSKY, F.—(1947) *Ibid.*, **4**, 396.

PAN, S. C., AND GARDNER, W. U.—(1948) *Cancer Res.*, **8**, 613.

ROSS, I. S.—(1939) *Arch. Path.*, **27**, 478.

SELBIE, F. R., AND THACKRAY, A. C.—(1948) *Brit. J. Cancer*, **2**, 380.

WELLS, H. G., SLYE, M., AND HOLMES, H. F.—(1941) *Cancer Res.*, **1**, 259.

WILLIS, R. A.—(1948) 'Pathology of Tumours.' London (Butterworth & Co., Ltd.), p. 367